# *Moringa oleifera* is a Prominent Source of Nutrients with Potential Health Benefits

**DOI:** 10.1155/2021/6627265

**Published:** 2021-08-10

**Authors:** Zahidul Islam, S. M. Rashadul Islam, Faruk Hossen, Kazi Mahtab-ul-Islam, Md. Rakibul Hasan, Rezaul Karim

**Affiliations:** Bangladesh Council of Scientific and Industrial Research (BCSIR), Dhaka 1205, Bangladesh

## Abstract

Nowadays, the socioeconomic status has been changed a lot, so people are now more concerned about their life style and health. They have knowledge about the detrimental effects of synthetic products. That is why they are interested in natural products. Utilization of natural products of plant origin having fewer side effects has gained popularity over the years. There is immense scope for natural products that can intimate health benefits beyond traditional nutrients. *Moringa oleifera* is one such tree having tremendous nutritional and medicinal benefits. It is rich in macro- and micronutrients and other bioactive compounds which are important for normal functioning of the body and prevention of certain diseases. Leaves, flowers, seeds, and almost all parts of this tree are edible and have immense therapeutic properties including antidiabetic, anticancer, antiulcer, antimicrobial, and antioxidant. Most of the recent studies suggested that Moringa should be used as a functional ingredient in food. The aim of this review is to focus the use of *Moringa oleifera* as a potential ingredient in food products.

## 1. Introduction

**Figure 1:**
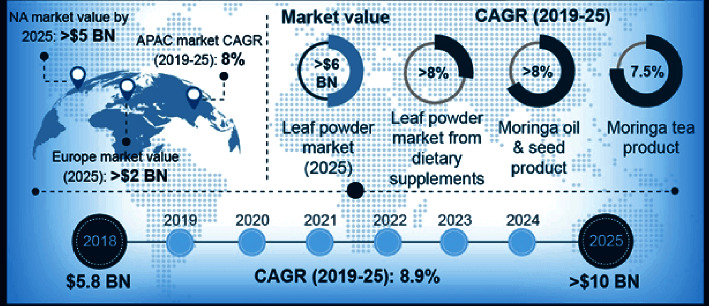
Moringa ingredient market.

In the context of global vision, one of the very first millennium development goals (steps to eradicate extreme hunger and poverty) is to “reduce by half the proportion of children that are malnourished (MDG).” Food deprivation is typically termed as hunger where malnutrition is something else. Malnutrition refers lack of quality food which is insufficient to support proper development and health. According to the report of the hunger project, in November 2017, 815 million people do not have food to sustain themselves out of 7.6 billion people of the world (Know Your World, The World Hunger Project). Moreover the exponential population growth threatens the margins of food security as time progresses. According to a report by the UN's Food and Agriculture Organization, earth is capable of ensuring the food security, where the agricultural sector needs a major transformation to reach its full potential [1]. *Moringa oleifera*, a native species of the Indian subcontinent, is a fast growing drought-resistant tree belonging to the family Moringaceae [2]. It is widely cultivated for the diversified use of its young seed pods and green leaves as vegetables and for medicine. It is considered as a very good supplement because of its high protein value. Without that, it is known as the miracle tree because of its diversified beneficial features, e.g., 10 times more vitamins than carrots, 7 times more vitamin C than oranges, 17 times more calcium than milk, and 15 times more potassium than bananas [3]. In addition, it helps to increase the blood antioxidant level [4] and reduce the blood sugar level [5] and sustained inflammation [6].  Figure 1 summarize a common scenario about global Moringa leaf powder market demand which is expected to exceed USD 6 billion up to 2025 on account of increasing demand in the dietary supplement and food applications [7].

**Figure 2:**
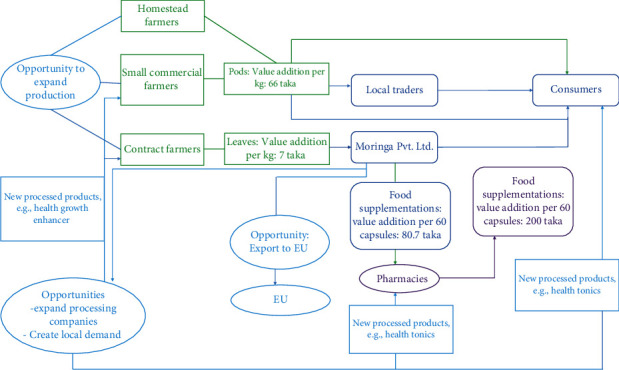
Market map of the current value chain and future opportunities in Bangladesh.

Most of the production and international trade of *M. oleifera* comes from India, in canned produce, fresh fruits, oil, seeds, and leaf powder [8]. India has an annual production of 1.1-1.3 million tons of tender pods [9]. According to Zauba.com, India exported Moringa leaf powder worth USD 4,746,132, equivalent to 836,806 kg for the period June 2013 up to July 2015. Of the total amount, the United States is the largest single buyer of Moringa leaf powder accounting for USD 3,303,870 followed by Germany and the United Kingdom which imported Moringa leaf powder worth USD 364,170 and USD 162,365, respectively. Netherlands imported Moringa leaf powder worth USD 12,594 over the same period [10]. On the other hand *Moringa oleifera* is not a crop of commercial interest in Bangladesh. Only consumption of pods is popular, and other uses such as leaves and flower consumption remain minimal. To improve the acceptability among the native people and the foreign market, different government and NGOs should come forward to explore the opportunities for export of Moringa.  Figure 2 illustrates the market diagram of Moringa-derived products and future opportunities in Bangladesh [11].

This study emphasizes on the importance of *Moringa oleifera* in food and nutraceutical industry and how it can assist in improving the health and well-being of humans. It focuses on the current status of *Moringa oleifera* as human food supplementation and future prospective.

### 1.1. Variety of Moringa Species and Its Geographical Distribution

The species Moringa is popular from the very ancient times because of its traditional use as a health curing agent and food as nutritionally enriched [12]. The single genus *Moringa* consists of 14 species [13] and 13 species that have been widely distributed and naturalized in Bangladesh, Sri Lanka, Pakistan, Arabian region, Africa, West Indies, Florida, South America, Peru, Paraguay, and Brazil [14] which is depicted in Table 1. *M. oleifera* is the most widely known and utilized of these [13]. The species of Moringa can be categorized into three groups on the basis of its trunk types [15].

The species from the plant list is taxonomically validated (http://www.theplantlist.org, v1.1, 2013). Among all these species, *Moringa oleifera* (Native in India and Bangladesh) has been cultivated in many countries of the world apart from the Pacific Islands, the Caribbean, Latin America, and Asia [14]. On the basis of the availability, *Moringa oleifera* was specified for this study for the clearance of specialty over other Moringa species. So much research has been done on this species (*Moringa oleifera*) since the 1970s [16]. Presently, the nutritional and medicinal importance is known to all.

### 1.2. Why Is Moringa known as the Miracle Tree?

The Moringa tree grows quickly, and they grow from seeds or cuttings of branch of trees. The tree leaves are something more than amazing though they grow quickly in poor soil within a very short period. Moreover, the tree is sustainable at dry and hot climates and is resistant to drought. The leaves, fruits, flowers, and immature pods of this tree are edible, and they form a part of traditional diets in many countries of the tropics and subtropics [17, 18]. Moringa is rich in nutrition owing to the presence of a variety of essential phytochemicals present in its leaves, pods, and seeds. In fact, Moringa is said to provide 7 times more vitamin C than oranges, 10 times more vitamin A than carrots, 17 times more calcium than milk, 9 times more protein than yoghurt, 15 times more potassium than bananas, and 25 times more iron than spinach [3]. The small leaves of Moringa pack a full punch of nutrients which contain more protein than eggs, more iron than spinach, more vitamin A than carrots, and more calcium than milk. The Moringa plant is found as a good source of energy with potential as pharmaceuticals and cosmetics (oils from seeds for hair and skin care) benefits. Moringa seeds are also rich in vitamins and minerals. Seed extracts show antibacterial activity and are also used as a water purifying agent. Various studies found Moringa seeds as oxidative stress-, inflammation-, blood sugar-, and blood pressure-reducing agents. People suffering from malnutrition and poverty found Moringa as a superfood because of its nutritional alternatives.

### 1.3. Food and Supplementation from *Moringa oleifera*

In the recent world, people are conscious but compelled to take calorific foods due to their busy schedule. Such food habits result in various health-related problems such as obesity, high blood pressure, diabetes, and various chronic diseases. For a balanced life style, a proper diet with an optimum level of vitamins, minerals, and PUFAs, etc. is required.

Moringa leaves are known as a very good food source which is easily digestible and rich in proteins [14]. According to Sultana and Anwar [19], Moringa leaves possess many valued compounds such as protein, vitamin, calcium, iron, ascorbic acid, and antioxidants (carotenoids, flavonoids, and phenol). Different developing or underdeveloped countries of the world feed their children with Moringa [20]. Busani et al. reported that the presence of numerous minerals and vitamins helps to improve the immunity against various diseases [21]. Moreover, Moringa leaves contain various amino acids. But nutrient variation is common because of climatic, location, and environmental factors [22]. Nowadays, Moringa leaves have diversified uses, such as medicinal coated capsules (as powder), as drinks (Ziga drinks), and tea [23]. Because of its nutritional properties, it is known as the miracle tree.  Table 2 depicts proximate profiles of Moringa fresh leaves, dry leaves, and leaf powder [24, 25].

### 1.4. Proteins and Amino Acids

Protein is an essential macronutrient for the human body responsible for overall growth, which is also known as a building block unit. Moringa leaves contain diversified phytochemicals such as sterols, tannins, flavonoids, alkaloids, saponins, and terpenoids [26]. Protein can be defined as a combination of long chain essential and nonessential amino acids which are linked by a peptide bond [27]. The essential amino acids need to be supplied externally as food items, whereas the nonessential amino acids can be produced by the body itself. Eggs, poultry meat, fish, red meat, etc. are known as common source of essential amino acids. Though it contains the maximum number of essential amino acids, it creates a problem for those people who are vegetarian, because most of the plant-derived protein do not contain the complete essential amino acid profile. In that case, the Moringa leaf powder could be a good alternative as a source of protein especially for essential amino acids. The American Dietetic Association and Dieticians of Canada reported that individuals who performed regular physical activities need 1.3 g-1.7 g protein per kg of body weight to repair body muscles.  Table 3 summarizes the protein percentage of total dry matter of different protein sources [28–32].

From the above table, it is clear that Moringa leaves contain a higher amount of protein. It is identified as alternative protein sources which can meet a regular demand of malnourished people [33]. People in many countries consume almost all parts of *Moringa oleifera* in various ways. Fresh Moringa leaves can be taken through cooking where the leaves can be stored as powder [14]. The seeds are also edible as it is green and dry [34]. However, Moringa leaves contain various types of amino acid. Amino acids such as Thr, Met, Ile, Lys, and Val were found to be present in Moringa leaves which are comparable to meat proteins [22]. Nowadays, Moringa leaf powder is incorporated in various valuable products.  Table 4 shows a comparison of the amino acid profiles of conventional animal and plant derived sources with Moringa leaf powder proteins [2, 31, 35].

In leaf source protein, the amino acid profiles are similar in the case of its availability. But the quality varies on the basis of species types, sources of raw materials, plant culture system, harvesting, and processing methods and analysis methods, etc. The miracle Moringa leaves contain 9 essential along with 7 nonessential amino acids.  Table 5 disseminates a comparative amino acid profile of Moringa leaf powder with some other consumable leaf powder [29–31].

### 1.5. Vitamins and Minerals

Besides macronutrients (carbohydrates, protein, and lipids), the animal body requires various micronutrients for survival. These micronutrients are very important for the body which act as a carrier or participate in the breakdown process of macronutrients. Vitamins are important which play a great role in energy processing of the animal body. Beriberi, rickets, scurvy, etc. are very common diseases which occur due to the deficiency of vitamins. Vitamins, such as vitamin A (beta-carotene), vitamin B (folic acid, pyridoxine, and nicotinic acid), vitamin C, vitamin D, and vitamin E, are found in *Moringa oleifera* [36]. Therefore, Moringa leaf powder or processed food derived from Moringa could be a good source of vitamins. Apart from various vitamins, Moringa contains lot of minerals, which are essential for physiological growth and development. Calcium is considered as one of the most important minerals, where dried Moringa powder is a great source of that element. It possesses 17 times more calcium than milk [22]. Without that, it contains 2 mg/100 g iron and 25.5-31.03 mg/kg zinc [22]. It is well enough to fulfill the daily requirement of zinc in the diet [37].  Table 6 depicts a list of vitamins and minerals found in leaf pods and seeds [24, 38].

### 1.6. Antioxidants

Normally human body maintains a balance ratio between oxidants and antioxidants. Because of external stresses in daily life, animal body continuously produces reactive oxygen species [39]. To balance the body with these free radicals, antioxidants are produced by the body cells. Any imbalance into these systems is known as oxidative stress. It can occur because of numerous diseases or imbalance in the normal physiological system [40]. In the severe stage, the oxidative stage turns the cell damage into various chronic diseases [41]. Kattappagari et al. [42] reported a positive feedback of antioxidant in such kind of chronic conditions that is preventing of further damages.

In terms of antioxidants, Moringa tree can be contemplated as a great source as it shows a higher production capacity than conventional plant-derived sources. Extracts from Moringa tree are capable of producing multiple types of components.

Table 7 comprises antioxidant activity of Moringa leaf methanolic extracts. Al-Taweel and Al-Anbari reported higher antioxidant activity (21.52%) which occurs due to its phenolic compound [43]. It is observed that Moringa leaf extract shows antioxidant activity both in vivo and in vitro due to its flavonoids and phenolic content [44, 45]. Methanol and ethanol extracts of *M. oleifera* leaves of Indian origin have the highest antioxidant activity with 65.1 and 66.8%, respectively [17, 46]. For those antioxidant properties, Moringa leaf powder can ensure protection against oxidative stress [47]. Bennett et al. reported chlorogenic acid, gallic acid, kaempferol and glycoside presence in Moringa leaves [48]. Freeze-dried Moringa leaves also show antioxidant activities [25]. However, for better supply for the consumers, naturally synthesized antioxidants have high demand.

## 2. Medicinal Uses

*Moringa oleifera* has numerous medicinal effective uses which have long been discerned in both the Ayurvedic and Unani systems [49]. Every part of *Moringa oleifera* is considered as important elements which have diversified medicinal value. Almost all parts of the Moringa trees have been used as natural medicine. Though Moringa tress extracts are used as a high valued food, besides it has various types of medicinal uses. Abalaka et al. [50] reported many pharmaceutical applications in the treatment of many diseases in the traditional medicinal system. In addition, Moringa aqueous extracts of roots were found significant in anti-inflammatory, antiulcer, and antitumor activities.

### 2.1. Antimicrobial and Anthelmintic Activities

Extracts from leaf, flower root bark, and stem bark of *Moringa oleifera* have antimicrobial and anthelmintic properties. Pterygospermin has powerful antibacterial and fungicidal activities [51] found by Das et al. and Rao et al. in the leaf and flower, respectively [52, 53]. Ethanolic extract of seeds, leaves, and flowers revealed the antimicrobial activity against *E. coli*, *P. aeruginosa*, *Enterobacter* species, *K. pneumoniae*, *S. aureus*, *Proteus mirabilis*, *Salmonella typhi* A, *Streptococcus*, and *Candida albicans* [54]. *Moringa Oleifera* flower and leaves have been demonstrated for their anthelmintic activity during several studies [55], for example, ethanolic extracts from *Moringa oleifera* leaves to inhibit Indian earthworm *Pheretima posthuma* [56].

### 2.2. Antiasthmatic Activity

Without showing any adverse effects with *M. oleifera* seed kernel, improvement was observed in the treatment of bronchial asthma patients and also their concurrent respiratory functions [57].

### 2.3. Anticancer and Antitumor Activity

There is a direct connection of Reactive Oxygen Species (ROS) with cell death. Various environmental stresses lead to excessive production of ROS causing progressive oxidative damage and ultimately cell death [58]. The compounds of the leaves that are held responsible for the anticancer activities are glucosinolates, niazimicin, and benzyl isothiocyanate. “Niazimicin” a bioactive compound from Moringa leaves showed potential anticancer activity [59]. Seven bioactive compounds, namely, 4(*α*-L-rhamnosyloxy)-benzyl isothiocyanate, niazimicin, 3-O-(6′-oleoyl-*β*-D-glucopyranoyl)-*β*-sitosterol, *β*-sitosterol-3-O-*β*-D-glucopyranoside, niazirin, *β*-sitosterol, and glycerol-1-(9-octadecnoate) had been isolated from the ethanol extract of the Moringa seed [60]. Benzyl isothiocyanate has been shown to be linked with cancer. Research showed that BITC causes intracellular ROS, which leads to cell death. This could be one of the reasons for Moringa to be a good anticancer agent [61–63]. Moringa contains an antiaging compound called Zeatin, which is a naturally occurring cytokinin [64] which has antitumor activities, effective against prostate and skin cancers, and is a strong antioxidant. The Moringa leaves also showed a significant cytotoxic effect on human myeloma cell lines [65].

### 2.4. Antidiabetic and Wound Healing Activity

Moringa is reported as an important element in controlling diabetes. Moringa leaves are reported as a significant agent in reducing blood glucose level immediately after taken [66]. The extracts (aqueous) from Moringa showed significant prohealing actions and a perfect wound healing characteristic [67].

### 2.5. Cardiac and Circulatory Stimulant and Antidiuretic Activities

The bioactive compound alkaloids from Moringa trees act as a cardiac stimulant [68] which are evident to stabilize blood pressure [60] influence on diuretic activity [13] and reduce fat and cholesterol [69] to prevent hyperlipidemia [70] and reduce serum triglyceride and serum cholesterols [71].

### 2.6. Analgesic Activity

Different parts of Moringa trees (leaves, pod, roots, etc.) showed analgesic activity. The alcoholic extract of Moringa leaves showed identical analgesic activity which was found through tail immersion method [72]. In another study, methanol extract of *M. oleifera* was tested in frog and guinea pig which showed that the plant (root bark) in both animals has produced significant local anaesthetic activity [73].

### 2.7. Antipyretic Activity

The antipyretic activity of Moringa was assessed in rats using different extracts (ethanol, petroleum ether, and ethyl acetate etc.) where seed extracts (ethanol and ethyl) showed significant activity [74].

### 2.8. Hepatoprotective Activity

The characteristics of protection against liver damage are reported about Moringa leaf extracts [75], and they also help in reducing liver fibrosis [76].

### 2.9. Antispasmodic and Antiulcer Effects

Because of the spasmolytic activity of Moringa trees, it is used traditionally to treat gastrointestinal motility disorder [77]. It is also reported that the methanolic extracts from this plant protects gastric lesions [78].

### 2.10. Other Medicinal Uses

*M. oleifera* pod constituents have bioactive compounds with anti-inflammatory activity which may contribute to ameliorate the pathogenesis of inflammatory-associated chronic diseases [79].Ovalbumin-induced airway inflammation in guinea pigs treated with n-butanol extract from *M. oleifera* seeds showed positive result as an anti-inflammatory agent [80]. Moringa was found to benefit and improve the condition against viral infectivity. Leaf extracts showed selective and potent inhibition of early steps in HIV-1 infectivity [81]. Hydroalcoholic extract of *M. oleifera* fruits showed antihepatitis B virus (HBV) activity [82]. Moringa is a potential source of vitamin A [83] which has a great potentiality to control the deficiency of vitamin A source food. Rather it could help to reduce eye problems of children.

### 2.11. Phytochemical Constituents of *Moringa oleifera* from Different Parts of the Plant

A number of medicinal properties have been described to various parts of this highly esteemed tree (Table 8). Almost all the parts of this plant, root, bark, gum, leaf, fruit (pods), flowers, seed, and seed oil, have been used for various ailments in indigenous medicines [84].

## 3. Market Trends

In recent times, people are aware about health-related issues, and they are very much interested in taking healthy and nutritious food. For instance, food which has a wide range of health benefits may prevent or cure various chronic diseases. This kind of extraordinary food is also termed as super food. It is a new field of research to identify such sources of healthy and nutritious foods.

The global Moringa markets are expected to increase significantly within the near future. According to MRFR analysis report, the global Moringa product field is expected to reach 9.3% which is USD 7902.9 million by 2025. Moringa products such as oils, capsules, leaf powder, and soaps are extracted from different parts of the tree. In contrast to the global contribution, the Asia Pacific contributed the largest share of 35.30% of the Moringa product market in 2018. Australia, China, India, and New Zealand are key countries which are contributing in the growth. Asia Pacific is the largest producer of Moringa. The production in the region is mostly consumed locally due to the traditional use of Moringa in wellness, skincare, and hair care. The market in North America is expected to register a CAGR of 10.0% by 2025.

## 4. Moringa Incorporation in Various Food Products

Moringa-derived food and nutraceutical products possess a huge potential which can decelerate the rate of malnourishment in developing nations. Some food products incorporated with leaf powder are shown in Table 9.

## 5. Risk Factors

We all know that Moringa is a nutritional miracle tree, but it has some side effects reported by Cadman [96].

1. *For pregnant women*: though Moringa leaves alone are enough to satisfy the daily iron and calcium need [22], it may possess antifertility characteristics in some cases
2. *For thyroid treatment*: though Moringa leaves aid thyroid function, it may create problems during the treatment with any other thyroid medication
3. *For diabetic medications*: though Moringa leaves effectively reduce blood sugar, it may cause too low blood sugar levels in some cases
4. *For blood pressure medication*: though Moringa is used in lowering blood pressure, Moringa with drugs that reduce blood pressure may result in a too low blood pressure

## 6. Conclusion

*Moringa oleifera* is a prominent source of nutrients and antioxidants. Like other vegetables such as spinach and fenugreek, Moringa leaves are not as popular all over the world, but currently, it is used as substitutes in soups, lentils, and other preparations in Southeast Asia. Still there is a knowledge gap in potential uses of Moringa as a food supplement and food fortification. Moringa has enormous potential uses but is very less explored. It can be utilized to make foods that could be a step towards curbing malnutrition. The published literature gives the total scenario of the chemical constituents, nutritional content, potential uses, and pharmacological activities of the plant. The identification, isolation, and standardization of plant extracts may be considered for detailed studies which can be useful for the further development of the promising food products with health benefits and nutrients to cure different life style-related diseases as well as malnutrition.

## Figures and Tables

**Table 1:** Categories of *Moringa* spp.

Category no.	Species	Categorization on the basis of trunk types	Geographical availability
1	*Moringa stenopetala*	Bottle tress (bloated water-storing trunks)	Kenya, Southwest Ethiopia, Somalia
	*Moringa drouhardii*		Southern Madagascar
	*Moringa ovalifolia*		Namibia, Southwest Angola
	*Moringa hildebrandtii*		Southern Madagascar
2	*Moringa peregrina*	Slender trunks	Red Sea, Arabia, Northeast Africa
	*Moringa concanensis*		India
	*Moringa oleifera*		India
3	*Moringa borziana*	Tuberous shrubs	Kenya, Somalia
	*Moringa arborea*		Kenya, Somalia
	*Moringa longituba*		Kenya, Southeast Ethiopia, Somalia
	*Moringa pygmaea*		North Somalia
	*Moringa rivae*		Kenya, Ethiopia
	*Moringa ruspoliana*		Kenya, Ethiopia, Somalia

**Table 2:** Proximate profiles of Moringa fresh leaves, dry leaves, and leaf powder.

Nutrients	g/100 g plant materials		
	Fresh leaves	Dry leaves	Dry leaf powder
Protein (g)	6.7	29.4	27.1
Fats (g)	1.7	5.2	2.3
Carbohydrate (g)	12.5	41.2	38.2
Fiber (g)	0.9	12.5	19.2

**Table 3:** Protein content in various plant leaves including Moringa.

Food origin	Protein content (% dry matter)
Rice	7.4
Wheat flour	12.1
Mulberry leaves (*Morus alba)*	20.88
Anchote leaves (*Coccinia abyssinica*)	21.6
Alfalfa leaves (*Medicago sativa)*	18.1
*Moringa oleifera*	
Fresh leaves	6.7
Dry leaves	29.4
Leaf powder	27.1

**Table 4:** Amino acid profile of different protein sources compared with Moringa leaf powder.

Source	Animal-derived sources					Plant-derived Sources			MLP^∗^
	Egg	CB	Rui fish	Pangas fish	Tilapia fish	SB	Rice	WF	
Essential amino acids (g/100 g of dry matter)									
Histidine	2.4	4.5	0.63	0.54	0.32	2.6	0.15	0.23	2.2
Isoleucine	6.6	3.24	0.76	0.62	0.76	5.3	0.23	0.31	4.8
Leucine	8.8	6.4	1.43	1.14	1.49	7.7	0.50	0.69	9.2
Lysine	5.3	7.9	1.58	1.25	1.60	6.4	0.23	0.28	5.6
Methionine	3.2	2.5	0.63	0.56	0.68	1.3	0.21	0.22	1.8
Phenyl-alanine	5.8	3.2	0.83	0.63	0.82	5.0	0.35	0.48	6.2
Threonine	5.0	3.7	0.87	0.68	0.9	4.0	0.22	0.30	4.2
Tryptophan	1.7	—	0.31	0.23	0.3	1.4	0.05	0.12	—
Valine	7.2	3.46	0.98	0.76	0.94	5.3	0.37	0.45	5.4
Nonessential amino acids (g/100 g of dry matter)									
Tyrosine	4.2	3.65	0.54	0.47	0.61	3.7	0.24	0.27	4.0
Alanine	—	4.7	2.19	1.63	2.22	5.0	0.57	0.56	6.3
Arginine	6.2	5.8	1.24	0.99	1.28	7.4	0.48	0.44	7.4
Asparagine	11.0	7.8	—	—	—	1.3	—	—	—
Glutamic acid	12.6	11.2	3.46	2.58	3.5	19.0	1.30	3.54	10.7
Glycine	4.2	3.4	1.36	2.58	3.49	4.5	0.29	0.41	5.3
Proline	4.2	3.2	0.99	0.75	0.89	5.3	0.27	1.07	2.9
Serine	6.9	3.4	0.80	0.63	0.81	5.8	0.32	0.51	4.1
Cystine	2.3	1.1	0.13	0.14	0.15	1.9	0.14	0.22	0.6

CB: chicken breast; SB: soybean; WF: wheat flour; MLP: Moringa leaf powder. ^∗^Amino acid profile of Moringa dried powder was analyzed at the Institute of Technology Transfer and Innovation (ITTI), Bangladesh Council of Scientific and Industrial Research (BCSIR), Dhaka.

**Table 5:** Comparative amino acid profile of different consumable leaf powders with Moringa leaf powder.

Source	Anchote leaves (*Coccinia abyssinica*)	Mulberry leaves (*Morus alba)*	Moringa leaf powder
Essential amino acids (g/100 g of dry matter)			
Histidine	1.63	3.56	2.2
Isoleucine	3.70	4.74	4.8
Leucine	5.38	6.89	9.2
Lysine	3.80	5.30	5.6
Methionine	0.93	1.78	1.8
Phenyl-alanine	3.10	3.34	6.2
Threonine	3.46	4.25	4.2
Tryptophan	—	—	—
Valine	4.17	5.16	5.4
Nonessential amino acids (g/100 g of dry matter)			
Tyrosine	1.63	11.42	4.0
Alanine	5.58		6.3
Arginine	4.33	4.52	7.4
Asparagine	8.94	8.26	—
Glutamic acid	8.92	—	10.7
Glycine	5.69	5.94	5.3
Proline	2.16	—	2.9
Serine	4.39	4.2	4.1
Cystine	3.27	—	0.6

**Table 6:** The vitamin and mineral compositions of leaves, seeds, and pods.

Nutrients	mg/100 g plant materials				
	Fresh leaves	Dry leaves	Powder	Seed	Pods
Vitamin B1	0.06	2.02	2.64	0.05	0.05
Vitamin B2	0.05	21.3	20.5	0.06	0.07
Vitamin B3	0.8	7.6	8.2	0.2	0.2
Vitamin C	220	15.8	17.3	4.5 ± 0.17	120
Vitamin E	448	10.8	113	751 ± 4.41	—
Calcium	440	2185	2003	45	30
Magnesium	42	448	368	635 ± 8.66	24
Phosphorus	70	252	204	75	110
Potassium	259	1236	1324	—	259
Copper	0.07	0.49	0.57	5.20 ± 0.15	3.1
Iron	0.85	25.6	28.2	—	5.3
Sulphur	—	—	870	0.05	137

**Table 7:** Antioxidant composition of *Moringa oleifera* leaf extract [43].

Parameters	Amount
Total flavonoids	257 mg/100 g quercetin equivalent
Total antioxidant capacity	1701.8 mg/100 g ascorbic acid equivalent
Total phenols	785.5 mg/100 g gallic acid equivalent

**Table 8:** Medicinal uses of different parts of the Moringa tree.

Parts	Uses	References
Leaves	Generally used for the treatment of asthma, bronchitis, hyperglycemia, dyslipidemia, flu, heart burn, syphilis, malaria, pneumonia, diarrhea, headaches, scurvy, skin diseases, eye and ear infections. Also it reduces, blood pressure and cholesterol, and it has anticancer, antimicrobial, antioxidant, antidiabetic, and antiatherosclerotic properties and it acts as a neuroprotectant agent	[3, 36], [85–87]
Seeds	Moringa seeds used for treating hyperthyroidism, Crohn's disease, antiherpes-simplex virus arthritis, rheumatism, gout, cramp, epilepsy, and sexually transmitted diseases, and they also act as antimicrobial and anti-inflammatory agents	[3, 20, 33], [88, 89]
Root bark	Used as a cardiac stimulant, antiulcer, and anti-inflammatory agent	[89, 90]
Flower	Used as a hypocholesterolemic, antiarthritic agents and can cure urinary problems and cold	[88]
Pods	Had a potential role for the treatment of diarrhea, liver and spleen problems, and joint pain	[38]

**Table 9:** Moringa incorporation in different food products.

Product types	Addition	Benefits	References
Soup	Alone or with spinach, melon, etc. As ingredient of soup	Increase the protein, fiber, and ash level into a satisfying amount	[91]
Moringa panner	Panner with extract of Moringa leaves		[92]
Chocolate	Moringa leaf powder as extra protein and fiber agent		[93]
Biscuits and cakes	Leaf powder in replacement of a percentage of flour		[93, 94]
Bread	Fortified with 5% leaf powder		[84]
Muffin	12% incorporation of leaf powder		[95]

## Data Availability

Previously reported article, case study, and report, etc. data were used to support this study and are available in the cited references. Only the amino acid values of the Moringa leaf powder amino acids and proximate data were analyzed at the Institute of Technology Transfer and Innovation, BCSIR, under the supervision of the authors. These prior studies (and datasets) are cited at relevant places within the text as references [1–97].
